# *In vivo* targeted single-nucleotide editing in zebrafish

**DOI:** 10.1038/s41598-018-29794-9

**Published:** 2018-07-30

**Authors:** Shingo Tanaka, Shin Yoshioka, Keiji Nishida, Hiroshi Hosokawa, Akira Kakizuka, Shingo Maegawa

**Affiliations:** 1 Department of Functional Biology, Graduate School of Biostudies, Kyoto University, Kyoto, 606-8501 Japan; 2 Graduate School of Science, Technology and Innovation, Kobe University, Kobe, Hyogo, 657-8501 Japan; 3 Department of Intelligence Science and Technology, Graduate School of Informatics, Kyoto University, Kyoto, 606-8501 Japan

## Abstract

To date, several genome editing technologies have been developed and are widely utilized in many fields of biology. Most of these technologies, if not all, use nucleases to create DNA double-strand breaks (DSBs), raising the potential risk of cell death and/or oncogenic transformation. The risks hinder their therapeutic applications in humans. Here, we show that *in vivo* targeted single-nucleotide editing in zebrafish, a vertebrate model organism, can be successfully accomplished with the Target-AID system, which involves deamination of a targeted cytidine to create a nucleotide substitution from cytosine to thymine after replication. Application of the system to two zebrafish genes, *chordin* (*chd*) and *one-eyed pinhead* (*oep*), successfully introduced premature stop codons (TAG or TAA) in the targeted genomic loci. The modifications were heritable and faithfully produced phenocopies of well-known homozygous mutants of each gene. These results demonstrate for the first time that the Target-AID system can create heritable nucleotide substitutions *in vivo* in a programmable manner, in vertebrates, namely zebrafish.

## Introduction

Genome editing technologies such as ZFN^1^, TALEN^2^ and the CRISPR/Cas9 system^3^ have become powerful tools for studying gene functions in model organisms, with a potential for gene therapy in humans^4^. Experimentally, therapeutic genome editing has been achieved in recent studies using technologies that involve non-homologous end joining (NHEJ)-mediated gene disruption^5–8^, NHEJ-mediated gene correction^9^, homology directed repair (HDR)-mediated gene correction^10–12^, and HDR-mediated gene addition^13^. These genome editing technologies have enormous potential for gene therapy. It should be kept in mind, however, that all of these technologies use nucleases to create DNA double-strand breaks (DSBs), and thus carry the risk of cell death and/or oncogenic transformation^14,15^. Therefore, DSB-free genome editing methods should be developed for therapeutic genome editing in humans.

We have recently created a novel single-nucleotide-editing technology that utilizes the CRISPR/Cas9 system for targeting without DSBs^16^. This technology is called the “Target-AID system”. In this system, a fusion protein consisting of nuclease-dead Cas9 (dCas9) or Cas9 nickase (nCas9(D10A))^3^, and the activation-induced cytidine deaminase (AID)^17^ derived from sea lamprey, PmCDA1, is introduced in cells with a single guide RNA (sgRNA). The sgRNA recruits the fusion protein, dCas9-PmCDA1, to target sites. AID tethered to dCas9 or nCas9(D10A) can deaminate cytosine in DNA within a five nucleotide window, resulting in the conversion of cytosine to thymine (C > T) after replication. The Target-AID system has been used to generate targeted C > T transitions in *Saccharomyces cerevisiae* and mammalian cultured cells^16^, but not yet in any vertebrates *in vivo*. This system has the potential to allow DSB-free genome editing in vertebrates, including humans, and might provide an alternative approach for gene therapy through single-nucleotide substitutions without DSBs.

Here we describe *in vivo* targeted single-nucleotide editing in zebrafish with the Target-AID system. The *chordin* (*chd*) and *one-eyed pinhead* (*oep*) genes were selected as target genes because the phenotypes induced by the loss (−/−) of each gene are well known. We first showed that the Target-AID system using dCas9-PmCDA1 or nCas9(D10A)-PmCDA1 induced targeted C > T substitutions in zebrafish embryos. Genomic analyses of F_1_ embryos produced by the founder fish (G_0_ fish), which had been co-injected with the dCas9-PmCDA1 mRNA and sgRNA, revealed that the targeted cytosines were substituted by thymines at appreciable frequencies. In addition, F_2_ fish with homozygous nucleotide substitutions in the target genes (*chd* or *oep*) manifested well-known mutant phenotypes. These results demonstrate for the first time that the Target-AID system produces programmable nucleotide substitutions in vertebrates *in vivo*.

## Results

### A strategy for *in vivo* nucleotide substitutions in zebrafish via the Target-AID system

**Figure 1:**
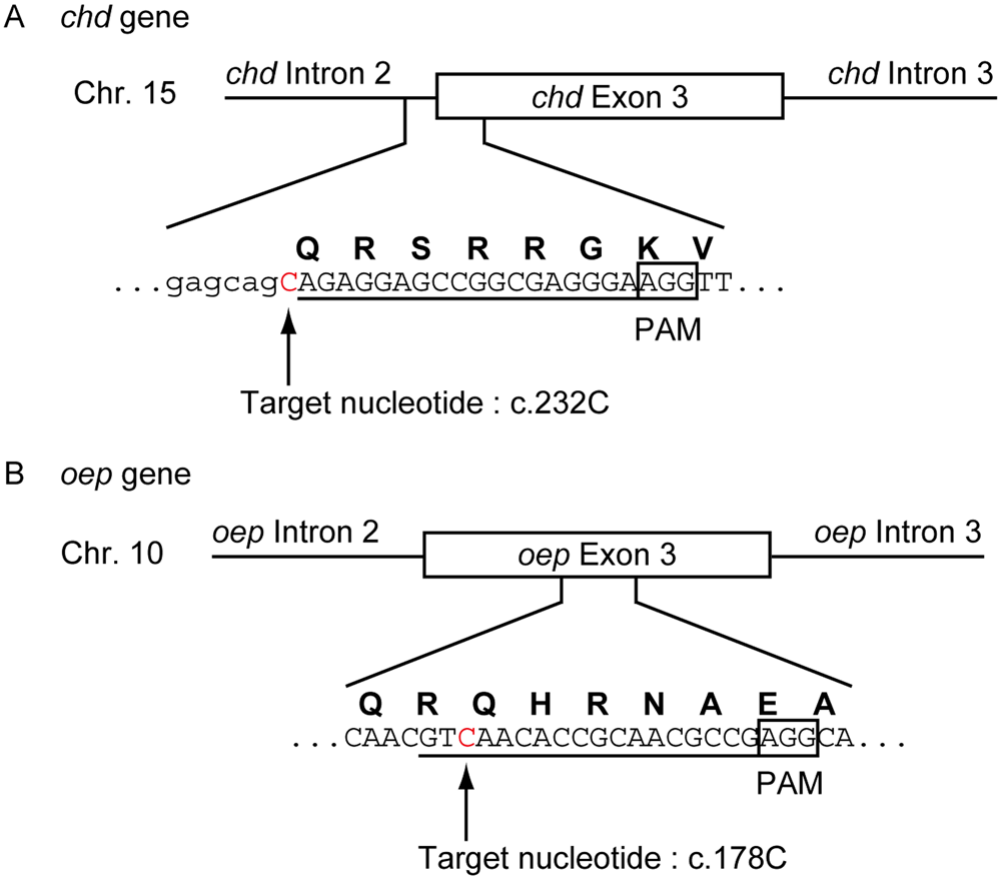
The design of the sgRNAs for the Target-AID system. Representations of the DNA sequences corresponding to the targeting sequences for (**A**) the *chd* sgRNA and (**B**) the *oep* sgRNA. (**A**,**B**) The uppercase letters in the DNA sequences represent exon sequences. The uppercase letters colored in red represent the target nucleotide. The lowercase letters represent intron sequences. The underlines represent the targeting sequences of the sgRNAs. The boxed sequences indicate the PAM sequence. The bold uppercase letters above the DNA sequences represent the single letter codes for amino acids.

In order to evaluate the feasibility of the Target-AID system in zebrafish, we first constructed a pCS2-based expression vector for *in vitro* synthesis of dCas9-NLS-FLAG-PmCDA1 mRNA (dCas9-PmCDA1 mRNA) and nCas9(D10A)-NLS-FLAG-PmCDA1 mRNA (nCas9-PmCDA1 mRNA). As targets of the Target-AID system, we selected two genes, the *chd* gene and the *oep* gene, because the phenotypes arising from the loss of each gene (−/−) have been thoroughly described. From our experiences with the CRISPR/Cas9 system, we were aware that single guide RNAs with 18 nucleotides targeting sequences work well^18^, and from the previous report for the Target-AID system, that cytosines between the -20 to -16 positions from the protospacer adjacent motif (PAM) sequence (5′–NGG for *Streptococcus pyogenes* Cas9 (SpCas9))^3^ are efficiently deaminated^16^. We therefore searched for cytosines in the *chd* and *oep* genes that were located -20 to -16 positions from potential PAM sequences, and whose changes to thymines would create stop codons (TAA, TAG, or TGA). Based on these criteria, we chose the 232^nd^ cytosine (c.232 C) in the coding sequence, located in exon 3 of the *chd* gene (Fig. 1A), and c.178 C, located in exon 3 of the *oep* gene (Fig. 1B), as the targets for the nucleotide substitutions. It is notable that deamination rates of cytosines located at the -20, -19, -18, -17, and -16 positions from PAM sequences are estimated to be roughly 5, 25, 45, 35, and 5%, respectively, based on the Target-AID system using nCas9(D10A)-PmCDA1 in yeast^16^. Thus, in the case of *oep* targeting, c.175 C (-19 position) was expected to be deaminated more efficiently than c.178 C (-16 position).

### Evaluation of the Target-AID system in zebrafish embryos

**Figure 2:**
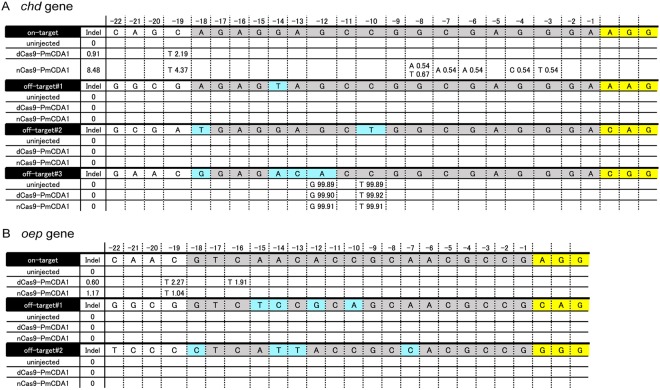
The Target-AID system can induce nucleotide substitutions at the target site in injected embryos. DNA sequences aligned in a row represent the reference sequences of target and off-target regions (DanRer10). The targeting sequences, mismatched bases, and PAM sequences in the DNA sequences are highlighted in gray, light blue, and yellow, respectively. Mutational frequencies (%) at each nucleotide position (-22 to -1) from the PAM sequences are shown below the DNA sequences. (**A**) A mutation spectrum in the *chd* locus obtained by deep sequencing for uninjected embryos, dCas9-PmCDA1 mRNA-injected embryos, and nCas9-PmCDA1 mRNA-injected embryos. (**B**) A mutation spectrum in the *oep* locus obtained by deep sequencing for uninjected embryos, dCas9-PmCDA1 mRNA-injected embryos, and nCas9-PmCDA1 mRNA-injected embryos. On average, more than 90,000 reads were obtained per sample.

To investigate whether the Target-AID system induced C > T substitutions at the target site in injected embryos, we injected dCas9-PmCDA1 mRNA or nCas9-PmCDA1 mRNA with *chd* sgRNA into zebrafish embryos at the 1-cell stage and incubated the embryos until 3 days post fertilization (dpf). We then analyzed sixteen uninjected and sixteen injected embryos, all of which showed wild-type phenotypes, by deep sequencing (Fig. 2A). The uninjected embryos had no mutation around the target site in the *chd* locus. The dCas9-PmCDA1 and nCas9-PmCDA1 mRNA-injected embryos contained targeted C > T substitutions at the -19 position (c.232 C) in the *chd* locus, with a frequency of 2.19% and 4.37%, respectively. Moreover, the nCas9-PmCDA1 mRNA-injected embryos contained C > A, G > T, G > C, or G > A substitutions with low frequencies; each of these substitutions was observed less than 1% in all samples sequenced. Of note, insertions and deletions (indels) were detected approximately 9 times less frequently in the dCas9-PmCDA1 mRNA-injected embryos (0.91%) than in the nCas9-PmCDA1 mRNA-injected embryos (8.48%), which is consistent with previous reports in yeast and cultured cells^16^. Heteroduplex mobility assays (HMA) also revealed that dCas9-PmCDA1 induced fewer indel mutations in the *chd* locus than nCas9-PmCDA1 and Cas9 (Suppl. Fig. 1A–C). These results demonstrate that the Target-AID system is able to induce C > T substitutions predominantly at the target site in zebrafish embryos.

Next, to examine if the Target-AID system produced off-target mutations in zebrafish, we performed deep sequencing for potential off-target sites. The potential off-target sites were predicted by CCtop software (http://crispr.cos.uni-heidelberg.de/index.html)^19^ based on the off-target mismatch score. We selected both of the potential off-target sites with the canonical (5′–NGG–3′) and non-canonical PAM sequences (5′–NAG–3′), as we previously reported^16^. With respect to the *chd* sgRNA, we analyzed three potential off-target sites. These potential off-target sites have 1- to 4-base mismatches from the true target site. Deep sequencing revealed that off-target#1 and off-target#2 contained no mutations in the uninjected embryos, none in dCas9-PmCDA1 mRNA-injected embryos, and none in nCas9-PmCDA1 mRNA-injected embryos (Fig. 2A). Off-target#3 had A > G and C > T substitutions at two positions (-10 and -12), both with almost 100% frequencies, which were most likely due to pre-existing single nucleotide polymorphisms rather than induced substitutions (Fig. 2A).

Next, we evaluated the *oep* gene locus (Fig. 2B). In each experiment, we analyzed sixteen embryos injected with *oep* sgRNA and dCas9-PmCDA1 mRNA, or sixteen embryos injected with *oep* sgRNA and nCas9-PmCDA1 mRNA, by deep sequencing. Collectively, as expected, targeted C > T substitutions were observed in the dCas9-PmCDA1 mRNA-injected embryos (2.27% at the -19 position (c.175 C), and 1.91% at the -16 position (c.178 C)). In the nCas9-PmCDA1 mRNA-injected embryos, targeted C > T substitutions were detected only at the -19 position (1.04%). Indel mutations were detected approximately 2 times less frequently in the dCas9-PmCDA1 mRNA-injected embryos (0.60%) than in the nCas9-PmCDA1 mRNA-injected embryos (1.17%). Similar to what we observed in the *chd* locus, HMA assays revealed that dCas9-PmCDA1 induced fewer indel mutations in the *oep* locus than nCas9-PmCDA1 and Cas9 (Suppl. Fig. 1D–F). Thus, the Target-AID system can induce targeted C > T substitutions with few indels in the *oep* gene locus in zebrafish embryos.

We next examined off-target effects in the *oep* locus. For the *oep* sgRNA, we analyzed two potential off-target sites. The potential off-target sites predicted by CCtop contained 4-base mismatches from the true target site. Deep sequencing revealed that both the potential off-target sites contained no mutations in the uninjected embryos, none in the dCas9-PmCDA1 mRNA-injected embryos, and none in the nCas9-PmCDA1 mRNA-injected embryos (Fig. 2B).

In summary, both constructs, dCas9-PmCDA1 and nCas9-PmCDA1, were able to induce targeted C > T substitutions in zebrafish embryos without apparent off-target effects, at least in the putative off-target sites. In particular, dCas9-PmCDA1 induced targeted C > T substitutions in both loci with fewer indel mutations than nCas9-PmCDA1 in zebrafish embryos. Importantly, the ratio of base substitutions to indel mutations^20^ with dCas9-PmCDA1 was higher than that with nCas9-PmCDA1 (in the *chd* locus, 2.41 for dCas9-PmCDA1 and 0.52 for nCas9-PmCDA1; and in the *oep* locus, 3.78 for dCas9-PmCDA1 and 0.97 for nCas9-PmCDA1). We therefore chose dCas9-PmCDA1 for the following experiments.

### Generation of G_0_ adult zebrafish carrying the expected nucleotide substitutions

We injected dCas9-PmCDA1 mRNA with the *chd* sgRNA or *oep* sgRNA into zebrafish embryos to generate G_0_ adult zebrafish (Suppl. Fig. 2). The adult fish are referred to as *chd* G_0_ fish or *oep* G_0_ fish, respectively. To investigate whether the G_0_ fish had the expected nucleotide substitutions at the target sites, PCR amplification of the targeted sequences was performed using genomic DNA from the caudal fins of five male *chd* G_0_ fish, and the PCR fragments were sequenced. Nucleotide substitutions were detected as extra peaks below the major peaks, likely due to mosaicism of the G_0_ fish (black and red arrowheads in Suppl. Fig. 3). In the analyses of *chd* G_0_ fish, two sequence patterns were obtained. One pattern, seen in three *chd* G_0_ fish, contained no detectable change (pattern (i) in Suppl. Fig. 3A); in the other pattern, two *chd* G_0_ fish had nucleotide substitutions at the target site, c.232 C (pattern (ii) in Suppl. Fig. 3A). No deletions were found around the target nucleotides in the *chd* gene. There were no detectable changes in non-targeted cytosines in the adjacent region around the target site in all analyzed *chd* G_0_ fish.

In the analyses of five male *oep* G_0_ fish, one fish had no detectable changes (pattern (i) in Suppl. Fig. 3B), and among the other fish, three types of nucleotide substitutions were observed (pattern (ii) to (v) in Suppl. Fig. 3B). The first type had a c.175 C > T substitution (black arrowheads in Suppl. Fig. 3B). The second type had a c.178 C > T substitution (red arrowheads in Suppl. Fig. 3B). In the third type, both c.175 C > T and c.178 C > T substitutions were present. Pattern (v), seen in one *oep* G_0_ fish, likely arose from mosaicism involving the expected nucleotide substitution of c.175 C and c.178 C, as well as at least one small deletion (Suppl. Fig. 3B). According to the experimental design, we expected that c.175 C and c.178 C, but no other nucleotide, would be mutated. Indeed, there were no detectable changes at other cytosines in the adjacent region around the target site in all analyzed *oep* G_0_ fish.

### The induced nucleotide substitutions were mostly heritable in the next generation

**Figure 3:**
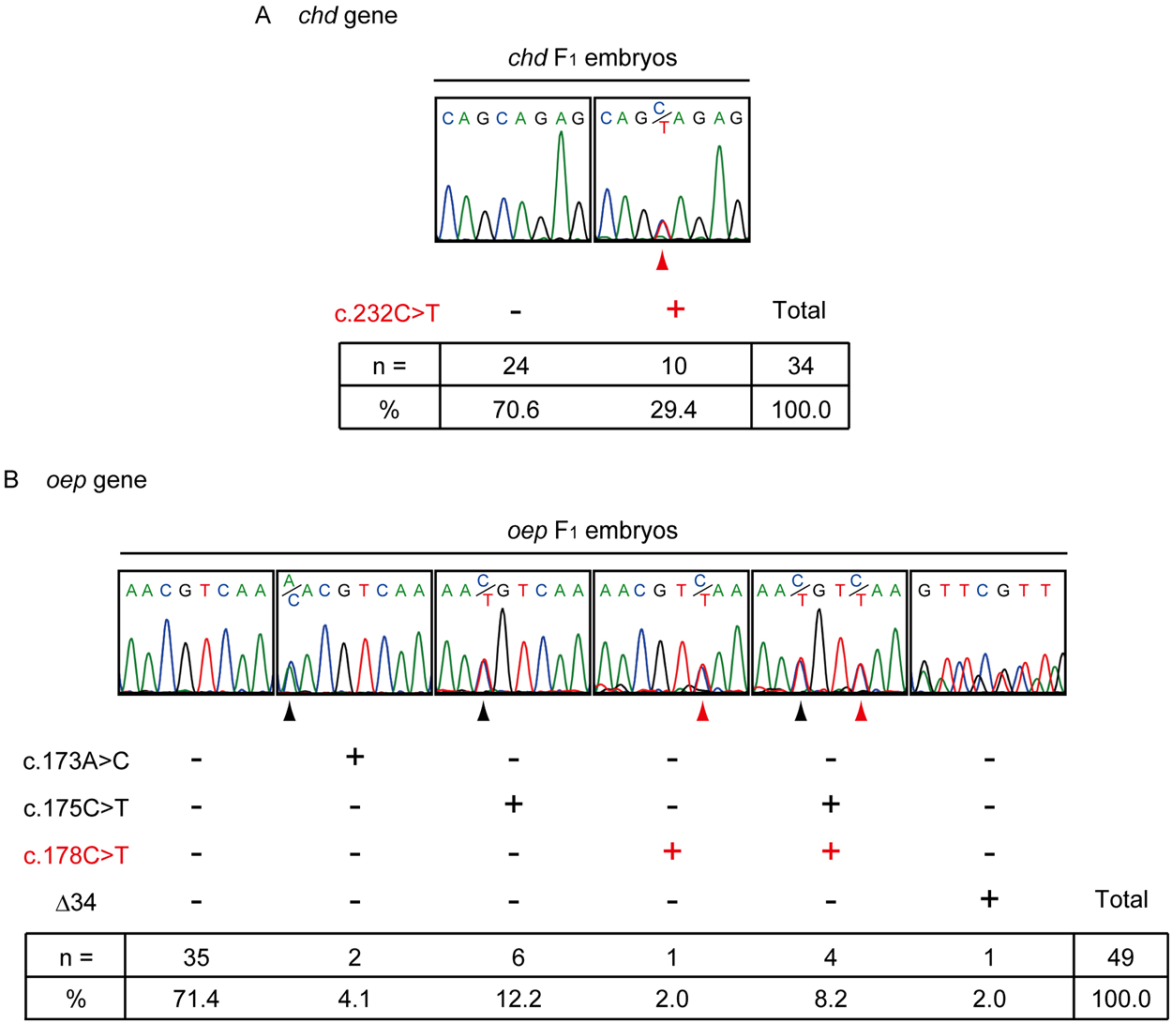
The nucleotide substitutions induced by the Target-AID system are heritable. Sequence patterns of (**A**) *chd* F_1_ embryos and (**B**) *oep* F_1_ embryos. The red arrowheads indicate nucleotide substitutions at the target site. The black arrowheads indicate nucleotide substitutions at untargeted sites. Observed frequencies and percentages of each genotype are shown in the panel.

In order to examine whether the nucleotide substitutions induced by the Target-AID system in the G_0_ fish were heritable, genotyping of descendant embryos of the G_0_ fish was performed. Wild-type females were bred with the *chd* G_0_ fish or *oep* G_0_ fish (Suppl. Fig. 2), and descendant embryos were obtained from three *chd* G_0_ fish and four *oep* G_0_ fish (*chd* F_1_ embryos and *oep* F_1_ embryos, respectively). We then performed PCR amplification of the targeted sequences using genomic DNA from F_1_ embryos and sequenced the PCR products. Nucleotide substitutions were detected as superimposed peaks because the F_1_ embryos were mostly heterozygous at the target loci (black and red arrowheads in Fig. 3).

Analysis of the genomic DNA from *chd* F_1_ embryos revealed two patterns in the *chd* loci (Fig. 3A). Expected c.232 C > T nucleotide substitutions were detected with a frequency of 29.4% (10/34). No detectable change was observed in the rest of the *chd* F_1_ embryos, 70.6% (24/34). Moreover, no other nucleotide substitutions were found within the sequenced regions. Germ line transmission of the modified allele was achieved in two of the three *chd* G_0_ fish (Suppl. Table 1).

From the analyses of *oep* F_1_ embryos from four *oep* G_0_ fish, we observed six patterns of sequence results (Fig. 3B): 1) 71.4% (35/49) of *oep* F_1_ embryos did not contain nucleotide changes; 2) 2.0% (1/49) contained a c.178 C > T substitution; 3) 12.2% (6/49) contained c.175 C > T substitutions; 4) 8.2% (4/49) contained both c.175 C > T and c.178 C > T substitutions; and 5) 2.0% (1/49) contained a deletion, detected in one sequencing result. Unexpectedly, in a sixth pattern two *oep* F_1_ embryos had a c.173 A > C substitution at a frequency of 4.1% (2/49). Three of the four *oep* G_0_ fish successfully transmitted the modified alleles (Suppl. Table 2). Note that one *oep* G_0_ fish with both the c.175 C > T and c.178 C > T substitutions (pattern (iv) in Fig. 3B) produced only wild-type F_1_ embryos (Fig. 3B and Suppl. Table 2). Taken together, these results indicate that the nucleotide substitutions induced by the Target-AID system are heritable.

**Figure 4:**
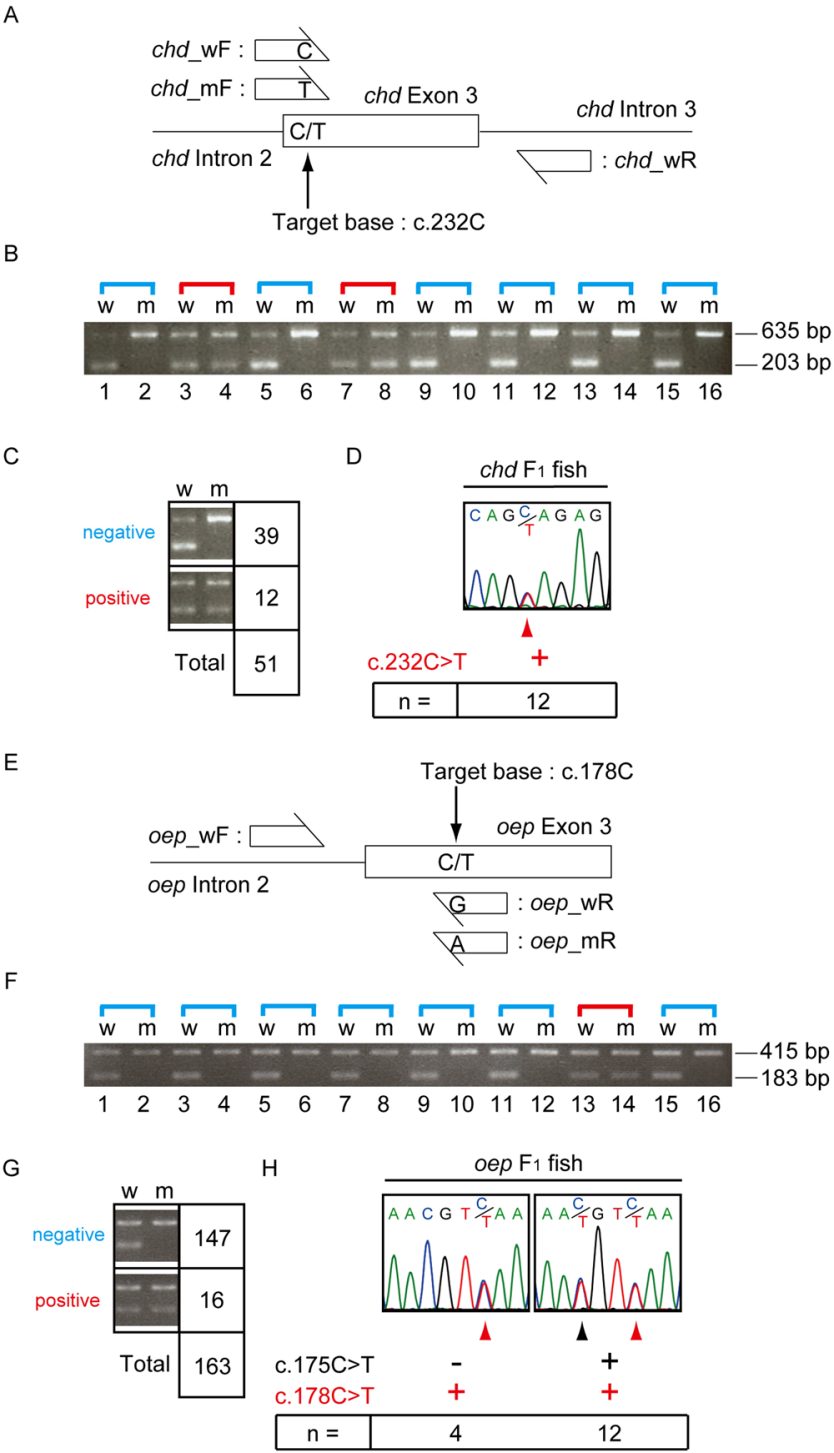
Identification of heterozygous fish carrying the expected nucleotide substitution at the target site. (**A**) Schematic representation of allele-specific PCR analysis of the *chd* gene. (**B**) An example of the results after allele-specific PCR amplification of the *chd* gene. The 203 bp DNA fragments in the”w” lanes were amplified by the primer set of *chd*_wF and *chd*_wR primers, whereas fragments in the “m” lanes were amplified by the primer set *chd*_mF and *chd*_wR. The 635 bp DNA fragments were amplified by a primer set recognizing a different region of the *chd* gene as an internal control. (**C**) Results of analyzing all individuals by allele-specific PCR for the *chd* gene. “Negative” indicates the absence of a nucleotide substitution at the target site, while “positive” indicates the presence of a nucleotide substitution at the target site. (**D**) Results of analyzing 12 positive individuals by sequencing the targeted region of the *chd* gene. (**E**) Schematic representation of allele-specific PCR analysis for the *oep* gene. (**F**) An example of results after allele-specific PCR amplification of the *oep* gene. The 183 bp DNA fragments in the”w” lanes were amplified by the primer set *oep*_wF and *oep*_wR, whereas fragments in the “m” lanes were amplified by the primer set *oep*_wF and *oep*_mR. The 415 bp DNA fragments were amplified by a primer set recognizing a different region of the *oep* gene as an internal control. (**G**) Results of analyzing all individuals by allele-specific PCR for the *oep* gene. (**H**) Results of analyzing 16 positive individuals by sequencing the targeted region of the *oep* gene. (**D**,**H**) The red arrowheads indicate the nucleotide substitution at the target site. (**H**) The black arrowhead indicates the nucleotide substitution at the untargeted site.

We next raised the rest of the F_1_ embryos to adulthood (Suppl. Fig. 2) and performed allele-specific PCR (Fig. 4A–C, E–G) and sequencing (Fig. 4D,H) to identify heterozygous F_1_ fish carrying the desired nucleotide substitutions at the target sites. For the identification of promising candidate heterozygous *chd* F_1_ fish carrying the c.232 C > T substitution (*chd*^c.232C>T/+^), we performed two PCR reactions using allele-specific primers, one for the wild-type allele (w) and the other for the mutated allele (m). Both produced 203 bp fragments (Fig. 4B,C). Another set of primers, which was designed to amplify the *chd* gene outside of the target site, was also included in the PCR reaction. This produced 635 bp DNA fragments as internal positive controls (Fig. 4B,C). When the 203 bp DNA fragment was detected only in a “w” lane, the result indicated that the fish did not carry the c.232 C > T substitution, and the fish was scored as a “negative” or “wild-type” fish (*chd*^+/+^ fish). If the 203 bp DNA fragments were detected in both “w” and “m” lanes, the fish was heterozygous, and was scored as “positive” or “mutated” (*chd*^c.232C>T/+^ fish). In total, we identified 39 “negative” (*chd*^+/+^) and 12 “positive” (*chd*^c.232C>T/+^) fish (Fig. 4C). Sequencing of the targeted region in the 12 positive fish confirmed the c.232 C > T substitution in all 12 fish (Fig. 4D).

We next examined *oep* F_1_ fish to identify candidate heterozygotes carrying the C > T nucleotide substitution (*oep*^c.178C>T/+^ fish) (Fig. 4E–G). For this purpose, we used sets of allele-specific primers for the wild-type allele (w) or for the mutated allele (m) (183 bp fragments in Fig. 4F,G), and another set of control primers (415 bp fragments in Fig. 4F,G), and performed allele-specific PCR. We found 147 (*oep*^+/+^) and 16 (*oep*^c.178C>T/+^) fish (Fig. 4G). Sequencing of the targeted region in the 16 (*oep*^c.178C>T/+^) fish revealed that 12 fish were compound heterozygotes with c.175 C > T and c.178 C > T substitutions, and 4 were heterozygotes for the c.178 C > T substitution only (Fig. 4H).

### The nucleotide substitutions at target cytosines recapitulate typical mutant phenotypes

**Figure 5:**
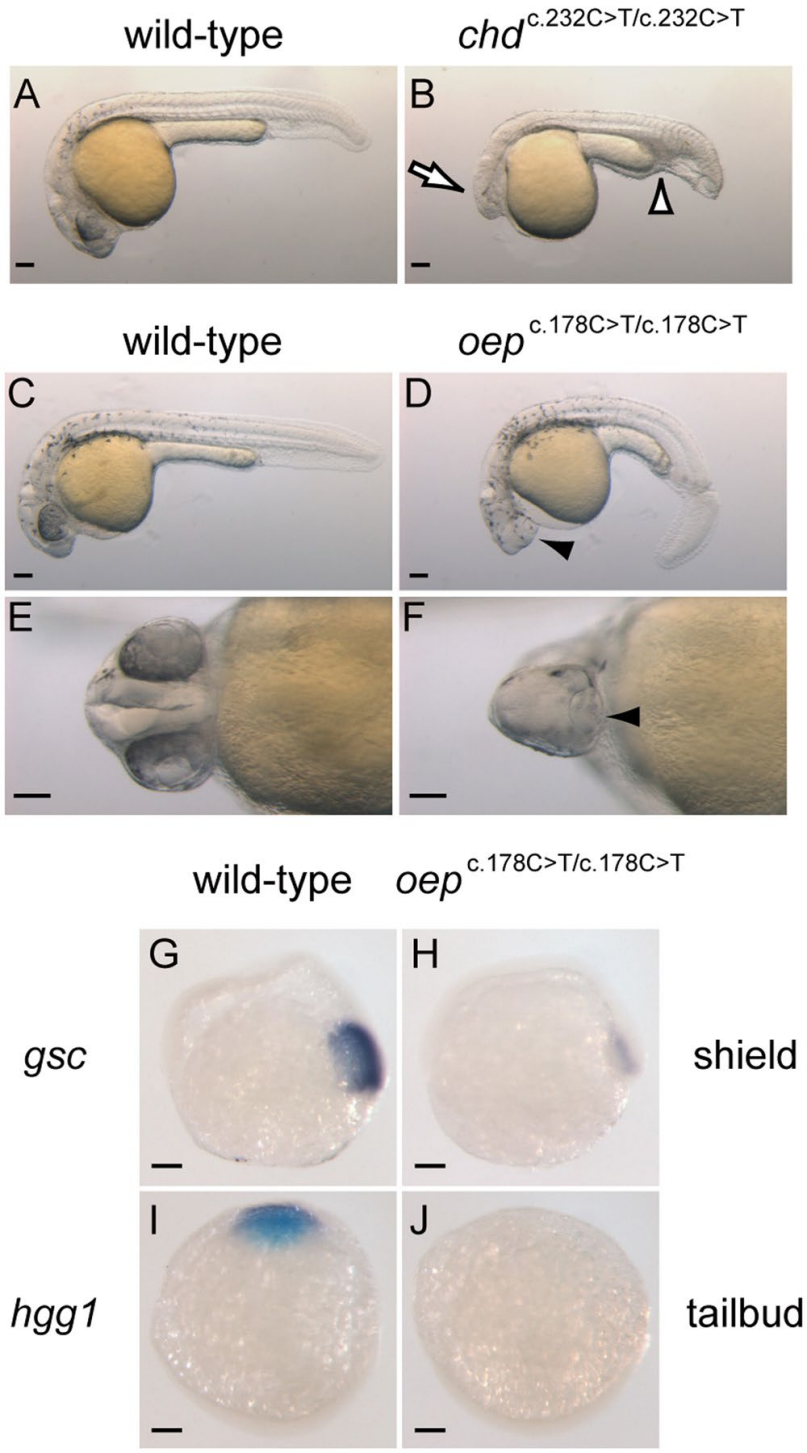
The Target-AID system can generate nucleotide substitution mutants. Phenotypic representation of (**A**, lateral view) a wild-type embryo and (**B**, lateral view) a *chd*^c.232C>T/c.232C>T^ F_2_ embryo at 24 hpf. The open arrow and open arrowhead indicate a smaller head and expanded blood islands, respectively. Phenotypic representation of (**C**, lateral view), (**E**, ventral view) a wild-type embryo and (**D**, lateral view), (**F**, ventral view) an *oep*^c.178C>T/c.178C>T^ F_2_ embryo at 27 hpf. The arrowhead indicates a single eye in each respective panel. (**G**,**H**) Expression pattern of *gsc* in (**G**, lateral view) a wild-type embryo and (**H**, lateral view) an *oep*^c.178C>T/c.178C>T^ F_2_ embryo at the shield stage. (**I**,**J**) Expression pattern of *hgg1* in (**I**, dorsal view) a wild-type embryo and (**J**, dorsal view) an *oep*^c.178C>T/c.178C>T^ F_2_ embryo at the tailbud stage. Scale bars represent 100 μm.

Next, we bred a pair of *chd*^c.232C>T/+^ fish or a pair of *oep*^c.178C>T/+^ fish to produce F_2_ embryos (Suppl. Fig. 2). As described above, the c.232 C > T and c.178 C > T nucleotide substitutions in the *chd* and *oep* genes, respectively, create premature stop codons (Fig. 1). Thus, the F_2_ embryos with homozygous mutations should display well-known phenotypes: *chd* mutants have small heads and expanded blood islands^21^, and *oep* mutants have single eyes^22^. The breeding experiments revealed that typical *chd* mutant and *oep* mutant phenotypes were observed in 28.4% (61/215) and 21.6% (68/315) of the F_2_ embryos, respectively. These results fit well with a recessive mode of Mendelian inheritance. Sequence analyses of the genomes of these embryos revealed the expected genotypes, namely F_2_ embryos with small heads and expanded blood islands were *chd*^c.232C>T/c.232C>T^ homozygotes (Fig. 5B compared with 5A), and F_2_ embryos with single eyes were *oep*^c.178C>T/c.178C>T^ homozygotes (Fig. 5D,F, compared with 5C,E, respectively).

To further characterize *oep*^c.178C>T/c.178C>T^ F_2_ embryos, we performed whole mount *in situ* hybridizations with RNA probes for *goosecoid* (*gsc*), a molecular marker for the organizer, and *hatching gland 1* (*hgg1*), a molecular marker for anterior dorsal mesoderm (Fig. 5G–J). The expression of *gsc* was suppressed at the shield stage in *oep*^c.178C>T/c.178C>T^ embryos (Fig. 5H compared with 5G). This observation was consistent with a previous study^22^. Moreover, the expression of *hgg1* was absent at the tailbud stage in *oep*^c.178C>T/c.178C>T^ embryos (Fig. 5J compared with 5I). These observations are also consistent with previously described phenotypes of *oep* mutants^22^, demonstrating that *oep*^c.178C>T/c.178C>T^ embryos recapitulate mesodermal defects characteristic of *oep* mutant embryos. These results, as a whole, convincingly show that the Target-AID system can introduce a targeted single-nucleotide substitution from cytosine to thymine *in vivo* in a vertebrate.

## Discussion

It has been shown that the Target-AID system^16^ and another nucleotide editing system^20^ deliver single-nucleotide alterations in *Saccharomyces cerevisiae* and mammalian cultured cells. The Target-AID system is based on the CRISPR/Cas9 system^16^ which functions as a powerful tool for gene knockouts in various kinds of vertebrates, such as medaka^18^, *Xenopus*^23,24^, and mouse^25,26^ as well as invertebrates^27,28^. Therefore, programmable single-nucleotide editing of genomic DNA in these various organisms should be amenable to the Target-AID system. Indeed, we show in this study that the Target-AID system can induce heritable nucleotide substitutions in zebrafish *in vivo*.

We evaluated two versions of the Target-AID system, dCas9-PmCDA1 and nCas9-PmCDA1, in zebrafish embryos. In a previous study of the Target-AID system, formation of nicks on the non-deamination strand of target DNA appeared to improve the frequency of C > T substitutions at the target sites^16^. Deep sequencing in injected embryos in the present study revealed that DNA nicking by nCas9-PmCDA1 improved the frequency of targeted C > T substitutions in the *chd* locus in zebrafish embryos, but not in the *oep* locus. Moreover, nCas9-PmCDA1 induced C > A, G > T, G > C, or G > A substitutions in addition to C > T substitutions in the *chd* locus, and increased indel mutations to a level that we consider problematic (Fig. 2 and Suppl. Fig. 1). In fact, indel mutations in zebrafish were observed in another nCas9-based base editing (BE) method, which induced C > T substitutions^29^. In sharp contrast, we observed efficient targeted C > T substitutions and hardly observed indel mutations with dCas9-PmCDA1, as compared to nCas9-PmCDA1. These results suggest that the Target-AID system using dCas9-PmCDA1 provides a safer single-nucleotide-editing method, as compared to nCas9-PmCDA1, for therapeutic applications in animals.

Deep sequencing of G_0_ embryos and analyses of F_1_ embryos revealed that the nucleotide substitutions at the target sites occurred with different frequencies. These differences may be dependent on the distances from the PAM sequences. With the *chd* gene, dCas9-PmCDA1 yielded the c.232 C > T mutation at the -19 position from the PAM sequence at a frequency of 2.19% in G_0_ embryos (Fig. 2A). With the *oep* gene, the c.175 C > T substitution (at position -19 with respect to the PAM) and the c.178 C > T substitution (at position -16 with respect to the PAM) were induced by dCas9-PmCDA1 in G_0_ embryos at frequencies of 2.27% and 1.91%, respectively (Fig. 2B). In F_1_ embryos, the *chd* c.232 C > T mutation was obtained at an overall frequency of 29.4% (10/34), and this was the sole mutation that was obtained (Fig. 3A). The c.175 C > T substitution was present in 20.4% of the *oep* F_1_ embryos, and the c.178 C > T substitution was present in 10.2% of the *oep* F_1_ embryos (Fig. 3B) when including compound heterozygotes with both -19 and -16 substitutions. These frequencies, in particular in F_1_ embryos, were reminiscent of those observed in a Target-AID system, using nCas9(D10A)-PmCDA1 in yeast^16^; with the yeast system, nucleotide substitutions of cytosines occurred at the -19 position at a frequency of about 25%, and substitutions at the -16 position occurred with a frequency of about 5%. Our results suggest that, even in zebrafish, the relative distance from the PAM affects the efficiency of cytidine deamination. Our results also indicate that candidate regions for targeted mutations should ideally have only one cytosine, in order to avoid redundant modifications.

Interestingly, base substitution rates in germ lines were higher than those of whole embryos in deep sequencing (Fig. 3A,B compared with Fig. 2A,B). Our observation suggests that base substitutions by dCas9-PmCDA1 might occur more effectively in germline cells than in somatic cells.

We also found A > C substitutions in the *oep* F_1_ embryos (Fig. 3B). The previous study reported that the A > C substitutions induced by the Target-AID system are rare (less than 1%)^16^. A deaminated adenine (hypoxanthine) is capable of base pairing with cytosine in *E. coli*, resulting in an A > G mutation^30^. However, this mechanism cannot explain the A > C substitution that we observed in the present study. A deletion event was also observed, even though nuclease-dead Cas9 was used for targeting (Figs 2, 3B, Suppl. Figs 1, and 3B). Deletion events with the Target-AID system using dCas9-PmCDA1 were also detected in the previous study at a frequency of less than 1%^16^. This deletion might be due to the sequence context in the targeted region and/or epigenetic modifications of the target loci. Further studies are needed for a detailed understanding of the rare A > C substitutions and deletions induced by the Target-AID system.

Off-target assessments in G_0_ embryos revealed that the Target-AID system did not appear to create off-target mutations, at least in the putative off-target sites we examined (Fig. 2). These results indicate that strict base-pair matching is required for the induction of C > T substitutions in zebrafish. Of course, there is a slight possibility that some of the injected embryos might die by 3 dpf because of unpredictable off-target effects. Further analysis about off-target effects of the Target-AID system is required to more precisely evaluate the Target-AID system *in vivo*.

G_0_ fish whose caudal fins possessed the desired nucleotide substitutions did not always transmit the nucleotide substitutions to their F_1_ descendants. The results are consistent with the possibility of mosaicism, where different nucleotide substitutions occurred in each daughter cell after the cell division of the zygote co-injected with the dCas9-PmCDA1 mRNA and sgRNA. The results also indicate that the genotype of cells in caudal fins is not a perfect indicator of germline transmission.

In addition to the creation of premature stop codons, as shown in this study, the Target-AID system has a potential to regulate transcription and splicing *in vivo*. It is difficult to make suitable precise modifications at promoter/enhancer regions or splicing sites by the regular CRISPR/Cas9 system because the CRISPR/Cas9 system induces uncontrolled DSBs. In contrast, the Target-AID system may allow researchers to modulate the promoter/enhancer activities and splicing pattern/efficiency by the substitutions of appropriate target cytosines with thymines in the promoter/enhancer and splicing sites, respectively. In this regard, the Target-AID system may provide an opportunity for therapeutic treatment by regulating gene expression and/or splicing. Future efforts will be directed toward overcoming the limitation of the PAM recognition, etc., to achieve nucleotide substitutions at any desired position of the genome.

## Methods

### Zebrafish

Zebrafish were maintained as described in a previous study^31^. Wild-type embryos were obtained by breeding AB strain males and females. All zebrafish experiments were performed under the ethical guidelines of Kyoto University and approved by the Animal Experimentation Committee of Kyoto University (No. Inf-K14001).

### mRNA synthesis

The DNA coding sequences for dCas9-NLS-FLAG-PmCDA1 (dCas9-PmCDA1), and nCas9-NLS-FLAG-PmCDA1 (nCas9-PmCDA1) were amplified by PCR with the following primer set: 5′-TCTTTTTGCAGGATCATGGACAAGAAGTAC-3′, and 5′-GAGAGGCCTTGAATTGGATCCTTATCCGGA-3′. The PCR products were cloned into the pCS2+ vector using the InFusion method (Takara, Kusatsu, Japan). The resulting pCS2+ dCas9-PmCDA1, the pCS2+ nCas9-PmCDA1, and the pCS2+ Cas9 plasmid was linearized with NotI. Capped mRNA encoding dCas9-PmCDA1, nCas9-PmCDA1, and Cas9 was synthesized *in vitro* from the SP6 promoter using a mMESSAGE mMACHINE SP6 kit (Thermo Fisher Scientific, Waltham, USA) following the manufacturer’s protocol.

### Single guide RNA synthesis

For synthesis of sgRNAs with customizable 18-nucleotide targeting sequences, we used the oligonucleotide pairs listed in Suppl. Table 3. Each oligonucleotide pair was annealed at 25 °C for 1 h after incubation at 95 °C for 2 min. The annealed oligonucleotides were ligated into BsaI-digested pDR274 vector^18,32^ with Ligation high Ver.2 (Toyobo, Osaka, Japan). After the ligated products were linearized with DraI, sgRNAs were synthesized *in vitro* using MEGAshortscript T7 Transcription Kit (Thermo Fisher Scientific, Waltham, USA) according to the manufacturer’s protocol.

### RNA injection

Approximately 1 nl of the following mixed solutions was microinjected into the cytoplasm of 1-cell stage zebrafish embryos: dCas9-PmCDA1 mRNA + *chd* sgRNA (100 ng/μl dCas9-PmCDA1 mRNA, 25 ng/μl *chd* sgRNA); nCas9-PmCDA1 mRNA + *chd* sgRNA (100 ng/μl nCas9-PmCDA1 mRNA, 25 ng/μl *chd* sgRNA); Cas9 mRNA + *chd* sgRNA (100 ng/μl Cas9 mRNA, 25 ng/μl *chd* sgRNA); dCas9-PmCDA1 mRNA + *oep* sgRNA (100 ng/μl dCas9-PmCDA1 mRNA, 25 ng/μl *oep* sgRNA); nCas9-PmCDA1 mRNA + *oep* sgRNA (100 ng/μl nCas9-PmCDA1 mRNA, 25 ng/μl *oep* sgRNA); and Cas9 mRNA + *oep* sgRNA (100 ng/μl Cas9 mRNA, 25 ng/μl *oep* sgRNA). For the *chd* sgRNA, on average, 96.6% of uninjected embryos, 77.8% of dCas9-PmCDA1 mRNA-injected embryos, 76.4% of nCas9-PmCDA1 mRNA-injected embryos, and 61.4% of Cas9 mRNA-injected embryos showed apparently normal phenotypes at 3 dpf. For the *oep* sgRNA, on average, 95.2% of uninjected embryos, 67.3% of dCas9-PmCDA1 mRNA-injected embryos, 57.6% of nCas9-PmCDA1 mRNA-injected embryos, and 47.2% of Cas9 mRNA-injected embryos showed apparently normal phenotypes at 3 dpf. Injected embryos with apparently normal phenotypes were used for deep sequencing and the heteroduplex mobility assays described below.

### Fin amputation

Adult zebrafish were anesthetized in 0.6 mM Tricaine solution (Sigma-Aldrich, St Louis, USA). Zebrafish caudal fins were amputated with a surgical blade (No. 10, FEATHER Safety Razor, Mino, Japan).

### Genomic DNA preparation

For genomic DNA preparation, amputated caudal fins of adult fish or whole single embryos at 1–3 dpf were lysed essentially as described^18^. The lysates were used for PCR amplification.

### Deep sequencing of target and off-target sites in zebrafish embryos

Genomic DNA, extracted from each zebrafish embryo at 3 dpf, was used as template DNA for primary PCR amplification. Deep sequencing was carried out as previously described^16^. The primers used for deep sequencing are listed in Suppl. Table 3. After nested PCR, index sequences were added to the amplicons. The index sequences were matched to samples as described in Suppl. Table 4. Sequencing reactions were performed with a MiniSeq sequencing system (Illumina, CA, USA) to obtain paired 151 bp read lengths. The obtained reads were mapped to each reference sequence from the zebrafish genome database (DanRer10) by the following setting: Masking mode = no masking; Mismatch cost = 2; Insertion cost = 3; Deletion cost = 3; Length fraction = 0.5; Similarity fraction = 0.8; Global alignment = No; Auto detect paired distances = Yes; Nonspecific match handling = Map randomly. The variant calling was performed with the following settings: Ignore positions with coverage = 150,000; Ignore broken pairs = Yes; Ignore Nonspecific matches = Reads; Minimum coverage = 10; Minimum count = 2; Minimum frequency = 0.5%; Base quality filter = No; Read detection filter = No; Relative read direction filter = 0.5%; Read position filter = No; Remove pyro-error variants = No. Rearrangement of the output file was made using Excel (Microsoft, WA, USA).

### Heteroduplex mobility assay

Heteroduplex mobility assays (HMA) were performed essentially as described in a previous study^18^. Targeting sequences in the *chd* and *oep* loci were amplified with BIOTAQ DNA Polymerase (Bioline, London, UK) and with primer sets listed in Suppl. Table 3. The primer sets produce 117 bp DNA fragments for the *chd* locus, and 132 bp DNA fragments for the *oep* locus. The DNA fragments were separated by electrophoresis in an 8.0% acrylamide gel.

### Genotyping by sequencing

PCR amplification of the targeting sequences in the *chd* and *oep* genes was performed with BIOTAQ DNA Polymerase (Bioline, London, UK) and with primer sets listed in Suppl. Table 3. PCR was carried out with the following parameters: for the *chd* gene, a pre-denaturation of 94 °C for 5 min, 30 cycles of amplification (94 °C for 20 s, 62 °C for 20 s and 72 °C for 20 s), and a final extension at 72 °C for 1 min; for the *oep* gene, a pre-denaturation of 94 °C for 5 min, 30 cycles of amplification (94 °C for 20 s, 55 °C for 20 s and 72 °C for 20 s), and a final extension at 72 °C for 1 min. The PCR products from the *chd* and *oep* amplifications were purified with NucleoSpin Gel and PCR Clean-up (Macherey-Nagel, Düren, Germany) and sequenced by Fasmac (Atsugi, Japan) or by Macrogen Japan (Kyoto, Japan) with the *chd*_1st_F and *oep*_1st_F primers, respectively.

### Genotyping by allele-specific PCR

Primers used in allele-specific PCR^33^ are listed in Suppl. Table 3. The *chd*_wF and the *chd*_mF primers were designed to anneal to the antisense strand of intron 2 and exon 3 in the *chd* gene, while the *chd*_wR primer to the sense strand of intron 3 in the *chd* gene. The 3′ ends of both of the forward primers were designed to anneal to the target nucleotide, c.232 C, in the *chd* gene. The *chd*_wF primer has a C residue at the 3′ end, while the *chd*_mF primer has a T residue at the 3′ end. Moreover, a T residue two nucleotides from the 3′ ends of both of the forward primers generates a template/primer C/T mismatch. The *oep*_wF primer was designed to anneal to the antisense strand of intron 2 in the *oep* gene, while the *oep*_wR and *oep*_mR primers to the sense strand of exon 3 in the *oep* gene. The 3′ ends of both of the reverse primers were designed to anneal to the target nucleotide, c.178 C, in the *oep* gene. The *oep*_wR primer has a G residue at the 3′ end, while the *oep* mR primer has an A residue at the 3′ end. The primer sets of *chd*_bF and *chd*_bR, and *oep*_bF and *oep*_bR were used for internal controls to amplify DNA sequences 800–1400 bp downstream of the target nucleotides.

Alelle-specific PCR for the target nucleotides in the *chd* and *oep* genes was performed with BIOTAQ DNA Polymerase (Bioline, London, UK) with the following PCR parameters: for the *chd* gene, a pre-denaturation of 94 °C for 5 min, 30 cycles of amplification (94 °C for 20 s, 60 °C for 20 s and 72 °C for 20 s) and a final extension at 72 °C for 1 min; for the *oep* gene, a pre-denaturation of 94 °C for 5 min, 25 cycles of amplification (94 °C for 20 s, 55 °C for 20 s and 72 °C for 20 s) and a final extension at 72 °C for 1 min. The PCR products were separated by electrophoresis in a 2.0% agarose gel.

### Phenotypic observation of zebrafish embryos

The phenotypes of F_2_ embryos were observed at 24 hours post fertilization (hpf) for the *chd* mutant embryos and at 27 hpf for the *oep* mutant embryos. Images of the embryos were taken under a SZX16 stereo microscope (Olympus, Tokyo, Japan) equipped with a MicroPublisher 5.0 camera (QImaging, Surrey, Canada).

### Whole mount *in situ* hybridization

Each RNA probe was synthesized to detect *gsc*, or *hgg1* endogenous mRNA, as described previously^34^. Whole mount *in situ* hybridization was performed by a protocol described previously^35^. Genomic DNA from each embryo was extracted essentially as described^36^. The genotype of each embryo was determined by allele-specific PCR.

### Data availability

Data obtained by deep sequencing have been deposited in the NCBI Sequence Read Archive (SRA), and the accession code is SRP140583. The remaining data are available from the corresponding author upon request.

## Electronic supplementary material

Supplementary information
