# Supplementary material for: In vivo targeted single-nucleotide editing in zebrafish
Source: Sci Rep. 2018 Jul 30;8:11423. doi: 10.1038/s41598-018-29794-9 (PMC6065354; doi:10.1038/s41598-018-29794-9)
Supplement: Supplementary file 1 — Supplementary information [file 41598_2018_29794_MOESM1_ESM.docx]

***In vivo* targeted single-nucleotide editing in zebrafish**

Shingo Tanaka^1^, Shin Yoshioka^2^, Keiji Nishida^2^, Hiroshi Hosokawa^3^, Akira Kakizuka^1^, and Shingo Maegawa^3^

**Supplementary Figure. 1.** dCas9-PmCDA1 induces fewer indel mutations than nCas9-PmCDA1. Heteroduplex mobility assay for the *chd* locus using genomic DNA from embryos injected with (A) dCas9-PmCDA1, (B) nCas9-PmCDA1, and (C) Cas9 mRNA. Heteroduplex mobility assay for the *oep* locus using genomic DNA from embryos injected with (D) dCas9-PmCDA1, (E) nCas9-PmCDA1, and (F) Cas9 mRNA. (A–C) The 117 bp fragment was amplified from the intact *chd* gene locus. (D–F) The 132 bp fragment was amplified from the intact *oep* gene locus. M: DNA size markers, NC: negative control, WT: uninjected wild-type, #1-#16: injected wild-type.

**
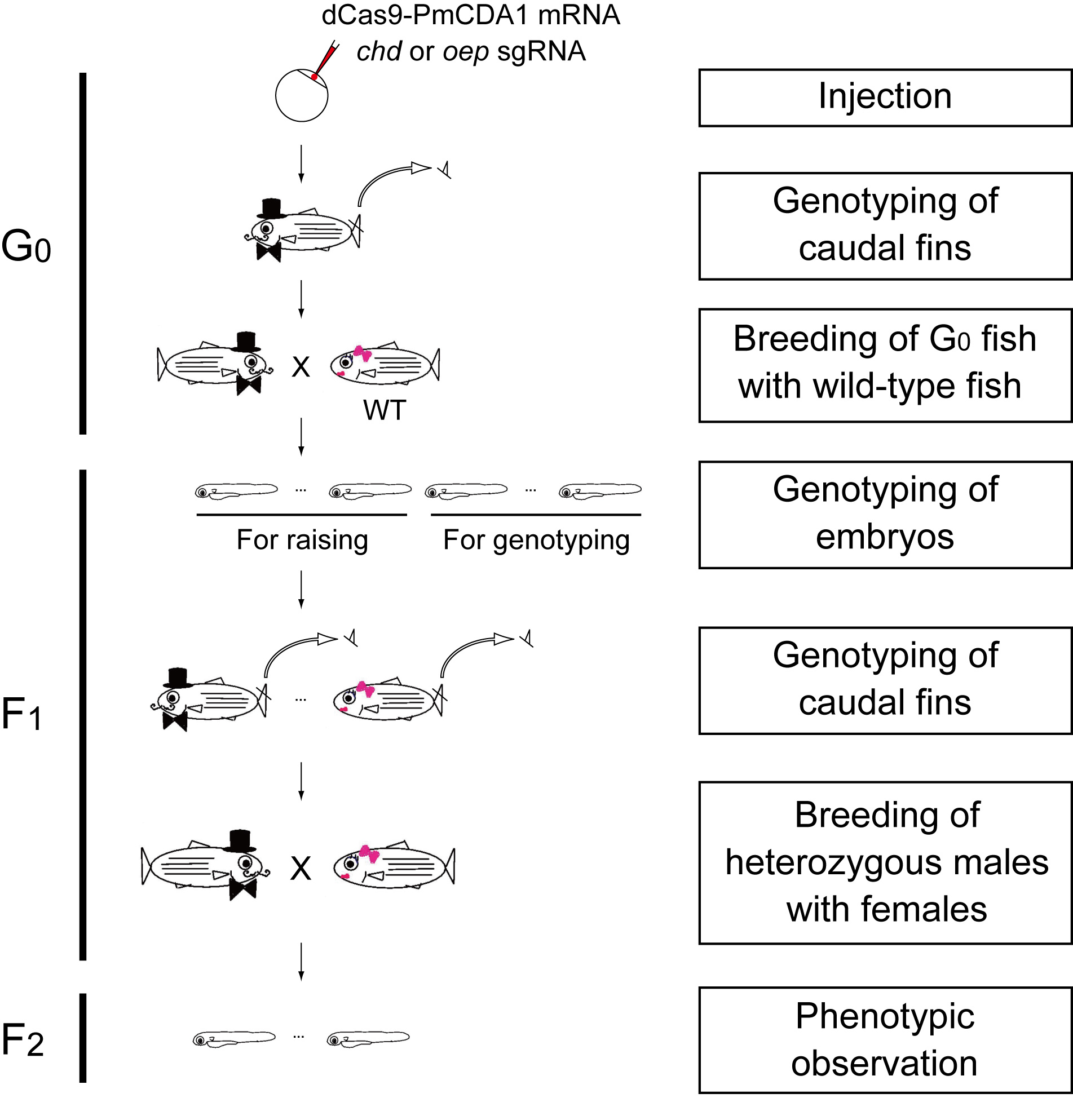
**

**Supplementary Figure 2.** A schematic diagram of the experimental procedures in this study. First, dCas9-PmCDA1 mRNA and *chd* or *oep* sgRNA were co-injected into zebrafish embryos just after fertilization. Five G_0_ fish per target gene were genotyped and separately bred with wild-type fish to obtain F_1_ descendants. F_1_ descendants were genotyped by allele-specific PCR and sequencing. Homozygous F_2_ embryos from mating two heterozygous fish were characterized with respect to morphology and molecular markers.


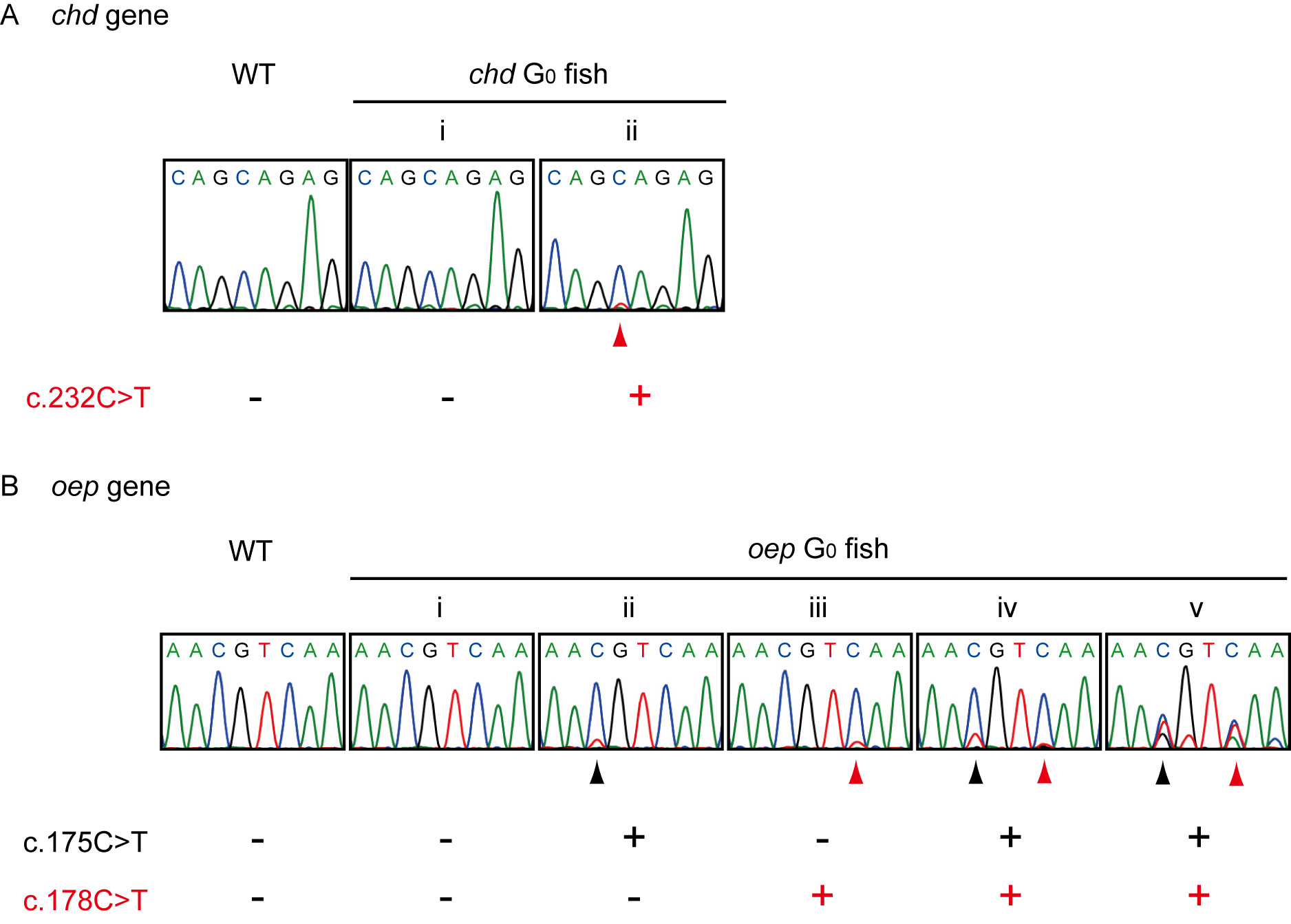

**Supplementary Figure 3.** The Target-AID system can induce nucleotide substitutions at the target site in G_0_ fish. Sequence patterns of (A) *chd* G_0_ fish and (B) *oep* G_0_ fish were obtained from genomic DNA of cells from excised caudal fins. The red arrowheads indicate nucleotide substitutions at the target site. The black arrowheads indicate nucleotide substitutions at untargeted sites.

**Supplementary Figure 4.** Full size gel images for Figure 4.

**Supplementary Table 1.** Distribution of each allele in *chd* F_1_ embryos produced by each *chd* G_0_ fish.

**Supplementary Table 2.** Distribution of each allele in *oep* F_1_ embryos produced by each *oep* G_0_ fish.

**Supplementary Table 3.** List of oligonucleotides and primers used in the present
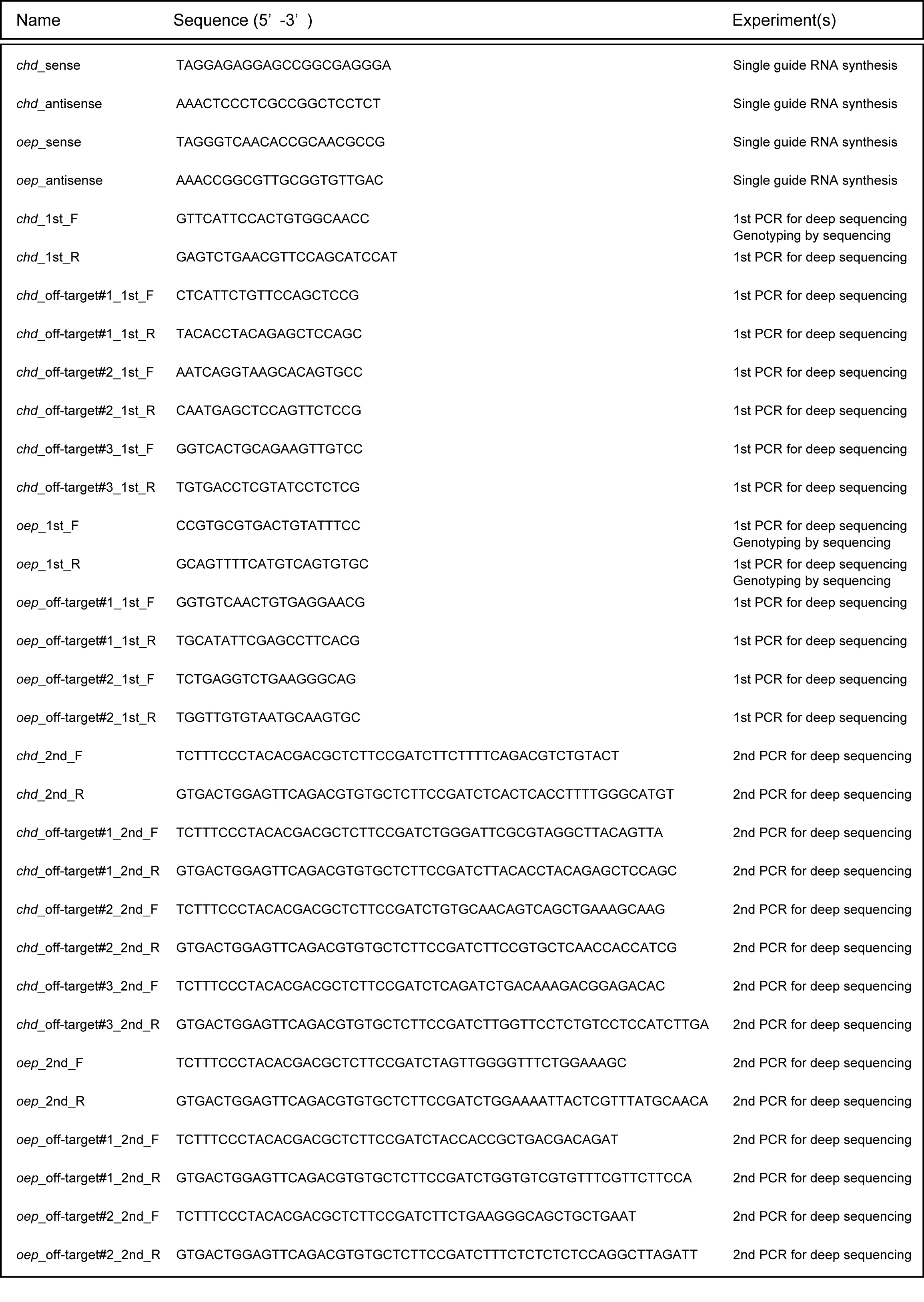
study.


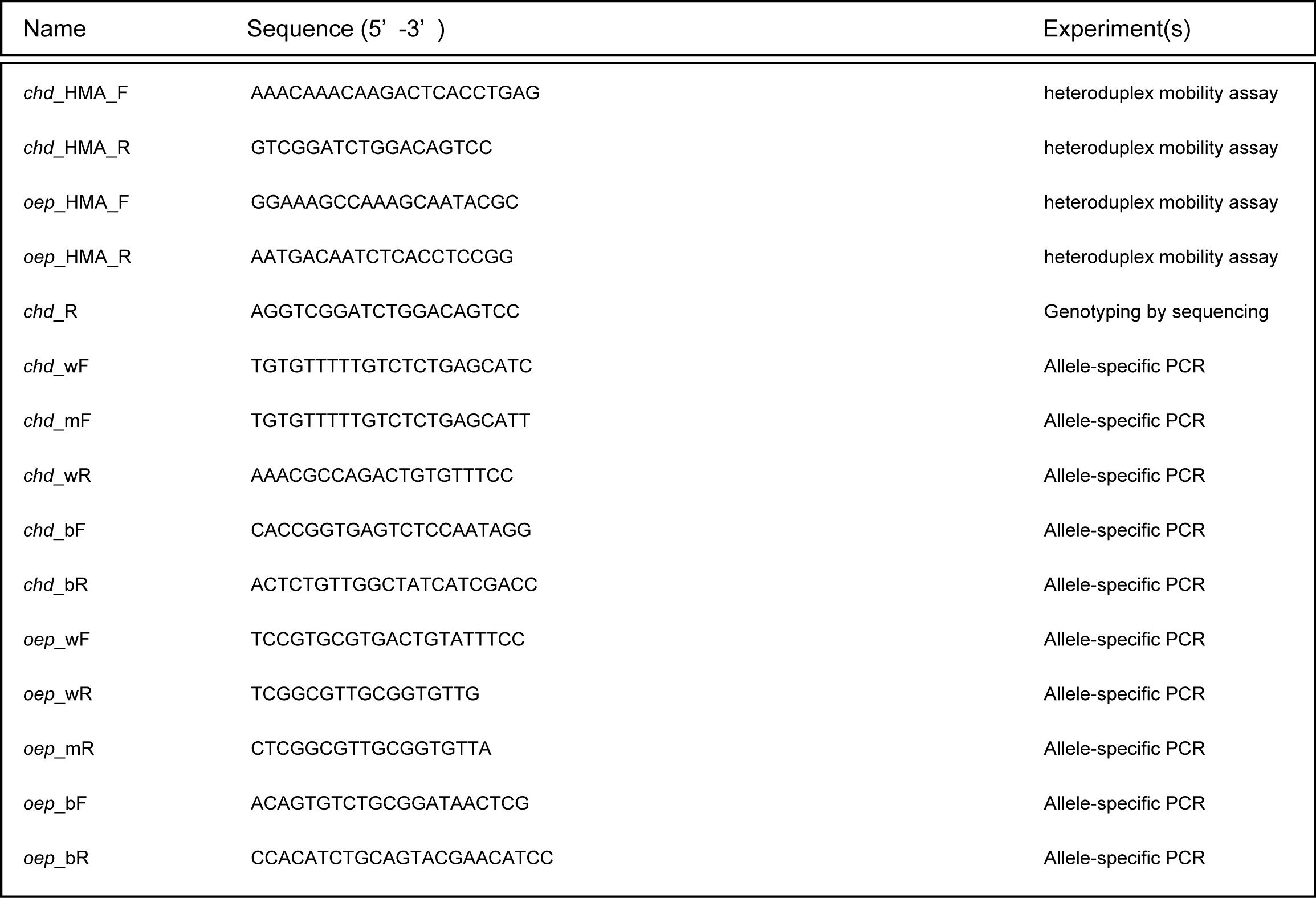

**Supplementary Table 4.** Index sequences and corresponding samples in deep sequencing.
